# Biodiversity and Ecosystem Function in the Gulf of Maine: Pattern and Role of Zooplankton and Pelagic Nekton

**DOI:** 10.1371/journal.pone.0016491

**Published:** 2011-01-31

**Authors:** Catherine L. Johnson, Jeffrey A. Runge, K. Alexandra Curtis, Edward G. Durbin, Jonathan A. Hare, Lewis S. Incze, Jason S. Link, Gary D. Melvin, Todd D. O'Brien, Lou Van Guelpen

**Affiliations:** 1 Bedford Institute of Oceanography, Fisheries and Oceans Canada, Dartmouth, Nova Scotia, Canada; 2 School of Marine Sciences, University of Maine and Gulf of Maine Research Institute, Portland, Maine, United States of America; 3 Acadia Centre for Estuarine Research, Acadia University, Wolfville, Nova Scotia, Canada; 4 Graduate School of Oceanography, University of Rhode Island, Narragansett, Rhode Island, United States of America; 5 Northeast Fisheries Science Center, National Marine Fisheries Service, Narragansett, Rhode Island, United States of America; 6 Aquatic Systems Group, University of Southern Maine, Portland, Maine, United States of America; 7 Northeast Fisheries Science Center, National Marine Fisheries Service, Woods Hole, Massachusetts, United States of America; 8 Saint Andrews Biological Station, Fisheries and Oceans Canada, Saint Andrews, New Brunswick, Canada; 9 Marine Ecosystems Division, Office of Science Technology, National Marine Fisheries Service, Silver Spring, Maryland, United States of America; 10 Atlantic Reference Centre, Huntsman Marine Science Centre, Saint Andrews, New Brunswick, Canada; University of California , Merced, United States of America

This paper forms part of a broader overview of biodiversity of marine life in the Gulf of Maine area (GoMA), facilitated by the GoMA Census of Marine Life program. It synthesizes current data on species diversity of zooplankton and pelagic nekton, including compilation of observed species and descriptions of seasonal, regional and cross-shelf diversity patterns. Zooplankton diversity in the GoMA is characterized by spatial differences in community composition among the neritic environment, the coastal shelf, and deep offshore waters. Copepod diversity increased with depth on the Scotian Shelf. On the coastal shelf of the western Gulf of Maine, the number of higher-level taxonomic groups declined with distance from shore, reflecting more nearshore meroplankton. Copepod diversity increased in late summer, and interdecadal diversity shifts were observed, including a period of higher diversity in the 1990s. Changes in species diversity were greatest on interannual scales, intermediate on seasonal scales, and smallest across regions, in contrast to abundance patterns, suggesting that zooplankton diversity may be a more sensitive indicator of ecosystem response to interannual climate variation than zooplankton abundance. Local factors such as bathymetry, proximity of the coast, and advection probably drive zooplankton and pelagic nekton diversity patterns in the GoMA, while ocean-basin-scale diversity patterns probably contribute to the increase in diversity at the Scotian Shelf break, a zone of mixing between the cold-temperate community of the shelf and the warm-water community offshore. Pressing research needs include establishment of a comprehensive system for observing change in zooplankton and pelagic nekton diversity, enhanced observations of “underknown” but important functional components of the ecosystem, population and metapopulation studies, and development of analytical modeling tools to enhance understanding of diversity patterns and drivers. Ultimately, sustained observations and modeling analysis of biodiversity must be effectively communicated to managers and incorporated into ecosystem approaches for management of GoMA living marine resources.

## Introduction

The biodiversity of a marine ecosystem plays an important role in its structure and function, and biodiversity information is increasingly used in management strategies for conserving harvested resources. Biodiversity comprises not only species variety, but also diversity in functional groupings and genetic variation within and among species [1]. All of these levels of biodiversity influence marine pelagic ecosystem interactions and processes, including primary and secondary production, nutrient cycling, and trophic transfer [2]. For ecosystem-based management of living marine resources (LMR), understanding biodiversity and dynamics of the pelagic ecosystem will inform conservation and harvesting decisions that affect marine mammal, fish and invertebrate abundance and diversity. Marine ecosystem management that incorporates understanding of biodiversity should lead to conservation of key species, augment resilience of process and function (i.e., functional redundancy, *sensu* [3]), enhance the capacity for marketing sustainable species (e.g., [4]), and facilitate the analysis of trade-offs among multiple resource uses. The zooplankton and pelagic nekton species of the Gulf of Maine are critically important to the function and structure of the region's ecosystem. A large amount of energy passes through pelagic organisms [5] [6], and zooplankton and pelagic nekton serve as a nexus between lower trophic level production and upper trophic level consumers that are of commercial, ecological, and aesthetic importance. Zooplankton and pelagic nekton serve as critical forage for a plethora of other species and often support targeted fisheries in their own right, for example herring [7]. Zooplankton and pelagic nekton package planktonic primary production into forms available for whales, pinnipeds, seabirds, fishes and humans [8] [9] [10] [11] [12]. Carnivorous zooplankton species are predators on and competitors for food of larval fish [13], establishing the potential for a cultivation-depensation loop [14]. Zooplankton and pelagic nekton thus contribute to a unique, highly connected system of interactions transferring energy within the Gulf of Maine pelagic food web [12] [15] [16].

**Figure 1:**
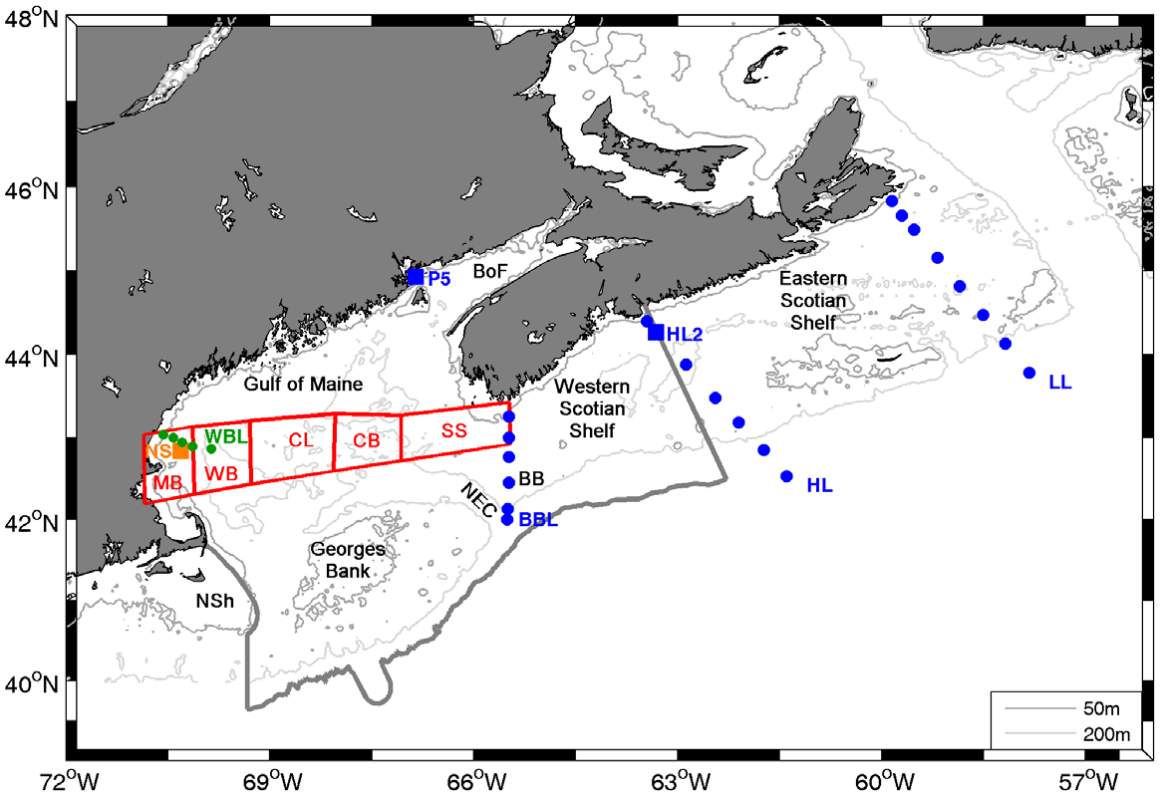
Gulf of Maine and Scotian Shelf, including areas sampled. Gray line indicates the boundary of the Gulf of Maine Area. Red boxes are regions sampled with the Continuous Plankton Recorder, and circles and squares are stations sampled with plankton nets. AZMP stations are indicated by blue symbols, COOA stations by green symbols, and PULSE by an orange symbol. BB – Browns Bank; BBL - Browns Bank Line; BoF - Bay of Fundy; CB - Crowell Basin; CL - Cashes Ledge; HL - Halifax Line; HL2 - Halifax line station 2; LL - Louisbourg Line; MB - Massachusetts Bay; NEC – Northeast Channel; NS - New Scantum (Jeffreys Ledge); NSh – Nantucket Shoals; P5 – Prince-5; SS - Scotian Shelf inflow; WB - Wilkinson Basin; WBL - Wilkinson Basin Line.

This paper examines the biodiversity of zooplankton and pelagic nekton in the Gulf of Maine Area (GoMA). As defined here, the GoMA includes the Gulf of Maine and Bay of Fundy, Georges Bank, the western Scotian Shelf, and the neighboring slope sea (Figure 1). The Gulf of Maine is a semi-enclosed sea, bounded by the coasts of Nova Scotia, New Brunswick, Maine, New Hampshire, and Massachusetts, and offshore by banks and shelves including Browns Bank, Georges Bank, and Nantucket Shoals. The region is bathymetrically complex and includes shallow banks and ledges and deep basins, the deepest of which is 377 m [17]. The dynamic pelagic habitat of the Gulf is also strongly influenced by its mean cyclonic circulation, with surface inflow of cold, lower-salinity water from the Scotian Shelf [18] and denser slope water through the Northeast Channel [19]. Slope water entering the Northeast Channel may be either colder Labrador Slope water or warm slope water [19]. Tidal mixing is very strong on the banks and in the eastern Gulf, especially in the Bay of Fundy. Primary production is high in the Gulf, particularly in coastal waters and on Georges Bank, and the spring phytoplankton bloom is a strong feature in the seasonal biological variability [20]. Biodiversity of zooplankton and pelagic nekton in GoMA is influenced by the diversity of pelagic habitats found in the region, which span a range of depth zones, temperatures, productivity levels, and mixing regimes. Immigration from upstream regions including the Scotian Shelf, continental slope and offshore waters also contributes to the biodiversity of the pelagic community.

We develop here a synthesis of the current knowledge of zooplankton and pelagic nekton species diversity and seasonal, regional, and cross-shore patterns by combining a review of past studies addressing diversity with new analysis of data. Comparisons of zooplankton diversity and communities over time and space have been hindered by differences in sampling designs and collection methods, as well as by limitations in sample analysis and access to data [21] [22]. We have made spatial and temporal comparisons only within consistently-sampled data sets but utilize multiple datasets to evaluate diversity over a range of spatial and temporal scales. Zooplankton studies in the GoMA have emphasized sampling of dominant mesozooplankton species (0.2 to 20 mm), and the focus of this paper is similarly based. Here, we primarily assess species diversity, but we also discuss diversity in terms of population and community structure and functional groupings. In the discussion, we consider principal drivers of diversity patterns and discuss functional roles of biodiversity. We also explore “underknown” taxonomic groups that may have significant roles in the GoMA ecosystem and put forward our collective perspectives on the most pressing questions and research needs for understanding zooplankton and pelagic nekton diversity, especially in the context of approaches to management of the region's ecosystem. The synthesis presented here is part of an overview of the biodiversity of marine life undertaken as part of the GoMA Census of Marine Life program. Complementary synthesis efforts are presented in this collection, including overviews for the benthos and demersal nekton, microbial communities, apex predators, coastal regions, and the continental slope and seamounts.

## Methods

We determined the overall number of named species of metazoan zooplankton, micronekton (*e.g.,* euphausiids, scyphozoans) and ichthyoplankton in the GoMA using the Gulf of Maine Register of Marine Species (GoMRMS) and several large databases containing zooplankton field sampling data. The unicellular plankton of the GoMA is discussed by Li et al. [23]. GoMRMS is a developing, authoritative list of marine species occurring in the GoMA, based on compendia and treatments of major groups or assemblages of organisms. This list is currently available at the Canadian Register of Marine Species website (http://www.marinespecies.org/carms/). It is a dynamic list that is being updated with missing, changed, and new records. Species names in the GoMRMS are being validated in terms of taxonomy and geography using published references, reliable web sources, or museum vouchers.

Species lists were also created from Fisheries and Oceans Canada (DFO) and the U.S. National Marine Fisheries Service (NMFS) plankton sample databases to describe the known alpha (within-community) diversity of metazoan zooplankton, micronekton and ichthyoplankton sampled in the GoMA. While the credibility of identifications in the databases is more variable than in the GoMRMS, the plankton sample data include more extensive zooplankton observational data than the data sources for the register. The Canadian data are served in the BioChem database (http://www.meds-sdmm.dfo-mpo.gc.ca/biochem/Biochem_e.htm), which includes plankton samples collected with a variety of net-based sampling systems using mesh sizes from 64–1179 µm, but mostly 100–300 µm, and with the continuous plankton recorder (CPR), which uses a standard nominal mesh size of 270 µm. The U.S. database (http://www.nefsc.noaa.gov/epd/ocean/MainPage/shelfwide.html) includes plankton samples collected with bongo nets equipped with 333 and 505 µm mesh during the MARMAP (Marine Resources Monitoring, Assessment, and Prediction) and EcoMon (Ecosystem Monitoring) programs as well as data from the NMFS CPR program. Ichthyoplankton data that contributed to the fish species list were obtained from the Northeast Fisheries Science Center [24].

Approximately 39,000 plankton net samples and 4,500 CPR samples have been collected and archived in the NMFS and DFO databases since 1961. Species names were validated and updated using the World Register of Marine Species (WoRMS) and the International Taxonomic Information Service (ITIS). Both of these sources are updated on a continuous basis (some sections are more up to date than others), and the species list reflects their status at the date when validation was performed. Following validation, the lists were reviewed by taxonomic analysts and researchers familiar with the regional plankton. The review identified two species, *Themisto gaudichaudii* and *Acartia clausi*, that were listed in the plankton databases prior to their redescription [25] [26]. They are valid species, but they do not live in GoMA waters and they were therefore removed from the list. Two abundant copepod species, *Pseudocalanus moultoni* and *P. newmani*, do not appear in either the GoMRMS or the species list. These species were often identified as *P. minutus* in the GoMA prior to taxonomic revision of the genus *Pseudocalanus* in 1989 [27], and they have not been identified to species in recent taxonomic analyses of NOAA and DFO monitoring samples due to their morphological similarity. Questions about the validity of several copepod species were resolved by reference to Razouls et al. [28], including the use of *Eurytemora affinis* in the species list rather than *E. hirundoides*. The species list generated from these databases was compared with GoMRMS to evaluate how much new information they provided, and a list of provisional additions to GoMRMS was made by combining the two lists. This list will remain provisional until vetted by experts. In addition, the *expected* diversity of ichthyoplankton (fish eggs and larvae) was calculated based on knowledge of the early life histories of fishes listed in the GoMRMS. The number of species that have pelagic, including bathypelagic, distributions as adults was also noted.

**Figure 2:**
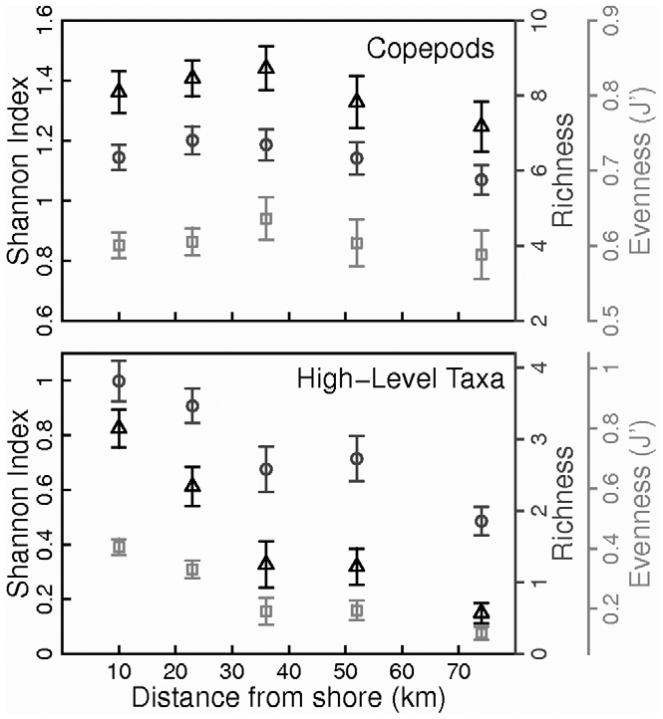
Cross-shelf variation in copepod and higher-level taxonomic diversity in the western Gulf of Maine. Triangles – Shannon index of diversity; circles - richness; squares - evenness. Data were collected and analyzed by the University of New Hampshire's Coastal Ocean Observing and Analysis program and C. Manning. Richness is based on rarefaction to 50 individuals. Bars indicate standard error, and N = 46, 45, 24, 25, and 19 for stations from nearshore to offshore, respectively. Higher level taxonomic groups were at classification levels ranging from class (e.g. Appendicularia and Polychaeta) to Phylum (e.g. Bryozoa and Cnidaria).

Numerous surveys of zooplankton have been conducted in the GoMA since the 1910s, but methodological differences in gear type, mesh size, and sampling depth, as well as geographical and seasonal differences in sampling effort and differences in taxonomic resolution among the sampling programs and over time make evaluation of long-term trends (50–100 years) in zooplankton biodiversity difficult or nearly impossible [29]. In the present study, spatial and temporal patterns in zooplankton diversity were described using data that were collected using comparable methods (i.e., within sampling programs and not between them) over 10 to more than 40 years. We used data from four programs. The University of New Hampshire's Center of Excellence for Coastal Ocean Observation and Analysis (COOA) sampled zooplankton monthly along a transect in the western Gulf of Maine from 2002 to 2007, using a ¼ m^2^ Multiple Opening and Closing Net and Environmental Sensing System (MOCNESS) equipped with 150 µm mesh (http://www.cooa.unh.edu/data/boats/zooplankton/; WBL stations in Figure 1). The PULSE Partnership for Pelagic Ecosystem Monitoring sampled weekly to semi-monthly in the western Gulf of Maine in 2003–2005 and 2007 ([30]; Jeffrey's Ledge Station NS in Figure 1). The Atlantic Zone Monitoring Program (AZMP) has sampled in the Canadian Maritimes region, including the Scotian Shelf and Bay of Fundy, twice yearly on broad-scale surveys (BBL, HL, and LL transects in Figure 1) and 1–2 times per month at two fixed stations since 1999 (P5 and H2 in Figure 1). Both PULSE and AZMP sample using vertical ring nets equipped with 202 µm mesh and towed from near-bottom to the surface [31]. The CPR survey, run by the Northeast Fisheries Science Center EcoMon Program in cooperation with the Sir Alister Hardy Foundation for Ocean Science, has sampled a monthly transect across the Gulf of Maine since 1961 (Figure 1). The CPR is towed at approximately 10 m below the surface and collects zooplankton on a continuous spool of 270 µm silk mesh [24]. Website sources and additional details about these programs are reported in Johnson & Hare [29]. In addition, National Marine Fisheries Service resource surveys were used to evaluate the interannual variability of small pelagic fish biomass [32] [33] [34].

Sample-based species diversity patterns were described using species richness, Shannon's H' diversity, and Pielou's J' evenness indices. Each monitoring program enumerated samples at multiple levels of taxonomic resolution. Here we report primarily on diversity patterns of adult copepods, which were most consistently identified to the species level. In the western Gulf of Maine, spatial variability in the diversity of higher-level taxonomic groups (e.g. at the order or class level) was also described. For the COOA and AZMP data, species richness in each sample was based on rarefaction to 50 individuals in order to correct for within-program differences in sampling effort. For CPR samples, richness was based on the number of species in each sample, which represents a standard sampling distance and volume. In the present context, richness should be considered as an index that was calculated consistently within each program but not between programs. Sample-based estimates of diversity indices may be biased by the small sample sizes enumerated, particularly for CPR samples, but these estimates represent a relative index of diversity that is comparable among samples collected using the same methods (i.e., within sampling programs).

For each sample, Shannon's H' diversity index was calculated as
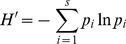
(1)where *S* is the number of species and *p_i_* is the proportional abundance of species *i*. Pielou's J' evenness index was calculated for each sample as
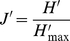
(2)where *H*'*_max_  = * ln*S*. Expected species accumulation curves were estimated and compared at stations sampled at monthly or more frequent intervals. Species richness, diversity indices, and expected species accumulation curves were calculated and plotted in R [35] [36] [37] [38] [39]. A two-way ANOVA with season and station as fixed factors was used to test for cross-shelf differences in the diversity of copepods and higher-level taxonomic groups in the western Gulf of Maine (WBL transect, Figure 1). Season was treated as a fixed factor to reflect distinct differences in the zooplankton production cycle during different times of year. A repeated measures ANOVA would not be appropriate for this case, because although sampling was repeated at the same geographic locations, different waters and more importantly different zooplankton assemblages were sampled during each station occupation due to along-shore advection.

Each of the three methodologies used here focuses on diversity at a different scale. The species register approach provides the broadest view in compiling a list of all species observed and reported in the region. In contrast, sample-based diversity estimates focus on patterns at short time and space scales. Expected species accumulation curves at repeatedly sampled fixed stations provide information about the number of species in the community over annual and interannual time scales. Together, these approaches provide an overall picture of the diversity of zooplankton and pelagic nekton in the region.

## Results

### Alpha diversity from GoMRMS and plankton samples

The plankton samples from the U.S. and Canadian databases contained 533 metazoan species, including 247 fishes and 237 crustaceans. Forty-seven percent of the species observed in plankton samples were not in GoMRMS (Table S1) and represent provisional additions to the register. Approximately half the additions are planktonic copepods, while other additions include fish (larvae and small myctophids), euphausiids, parasitic copepods, larvaceans, opisthobranch mollusks, and chaetognaths, followed by a number of orders with only a single “new” species each. Some of the species are common and abundant, and their addition here is more the result of “uncovery” than “discovery.” Their absence from the register up to this time reflects prior register emphasis on demersal fish and benthic invertebrates, greater sampling effort on the shelf, and less effort at data mining specifically to assess species richness. We note that many of the ‘new’ copepods, euphausiids and fishes came from the outer shelf and slope.

Biphasic life histories are typical of the majority of marine benthic invertebrates, of which there are over 2000 named species in the GoMA. Early life development and dispersal strategies are highly variable, however, and descriptions are lacking for a large number of taxa. Doubtless, there are many meroplanktonic species that were not identified here. The largest numbers would be expected among the annelids, crustaceans, echinoderms and mollusks—speciose groups for which planktonic stages are common.

More is known about the fishes in the GoMA than the invertebrates in terms of their distribution, abundance, and life histories. In Table 1 we list the number of fish species by order and examine their reproductive strategies to gauge how many species might be contributing eggs and/or larvae to the diversity of plankton. Comparison of the expected ichthyoplankton diversity with ichthyoplankton species observed in plankton samples revealed a few species not listed in GoMRMS. There are currently 497 species of fishes in GoMRMS, of which 352 (71%) have been validated. Species with adults that are pelagic or benthopelagic number 289 (58%), leaving 209 benthic or demersal species (42%). Eighteen fish species are anadromous, three are catadromous, and three are amphidromous, totaling 24 diadromous species (5%). While the early life history remains unknown for 86 species (17%), we can identify that 356 (72%: Table 1) produce planktonic stages (eggs and/or larvae), while 55 (11%) do not. Thus, the U.S. and Canadian sample data (247 species) do not represent the full expected diversity of ichthyoplankton, most likely due to difficulties with identification, seasonality, low abundance, or spawning/nursery areas with little or no sampling for the databases we analyzed.

**Table 1:** Ichthyoplankton in the Gulf of Maine Area.

		GoMRMS			Sample Data	Provisional Additions
Class	Order	# Species	# Known mero-planktonic species	Life history of remaining species	# Species	# Species
Actinopterygii	Acipenseriformes	2	2			
	Albuliformes	1	1			
	Aulopiformes	22	21	1 unknown	14	5
	Anguilliformes	25	20	5 unknown	17	5
	Atheriniformes	2	2		1	
	Batrachoidiformes	1	1			
	Beloniformes	9	9		1	
	Beryciformes	8	5	3 unknown	1	1
	Cetomimiformes	2	1	1 unknown	1	1
	Clupeiformes	11	11		8	2
	Cyprinodontiformes	3	3			
	Elopiformes	2	2		2	
	Gadiformes	28	25	3 unknown	14	3
	Gasterosteiformes	4	3	1 unknown	2	
	Lampriformes	1	1			
	Lophiiformes	19	13	6 unknown	1	
	Myctophiformes	31	27	4 unknown	34	11
	Notacanthiformes	5	0	5 unknown		
	Ophidiiformes	4	4		6	3
	Osmeriformes	12	9	3 unknown	5	1
	Perciformes	127	105	21 unknown, 1 nonplanktonic	66	16
	Pleuronectiformes	24	21	3 unknown	23	6
	Polymyxiiformes	2	1	1 unknown		
	Saccopharyngiformes	1	1			
	Salmoniformes	7	0	7 nonplanktonic		
	Scorpaeniformes	32	23	9 unknown	20	2
	Stephanoberyciformes	6	5	1 unknown		
	Stomiiformes	35	24	11 unknown	22	7
	Syngnathiformes	8	4	4 unknown	4	
	Tetraodontiformes	12	10	2 unknown	4	
	Zeiformes	4	2	2 unknown		
Cephalaspidomorphi	Petromyzontiformes	1	0	1 nonplanktonic		
Elasmobranchii	Carcharhiniformes	13	0	13 nonplanktonic		
	Hexanchiformes	1	0	1 nonplanktonic		
	Lamniformes	6	0	6 nonplanktonic	1	
	Rajiformes	16	0	16 nonplanktonic		
	Squaliformes	8	0	8 nonplanktonic		
	Torpediniformes	1	0	1 nonplanktonic		
Holocephali	Chimaeriformes	1	0	1 nonplanktonic		
	**Total**	497	356	86 unknown	247	63
				55 nonplanktonic		

Ichthyoplankton species numbers, including life history information, from the Gulf of Maine Register of Marine Species (GoMRMS) and plankton samples. Meroplanktonic stages may be eggs and/or larvae. Provisional additions are species in the samples that were not in GoMRMS.

A list of all planktonic metazoan species presently identified in the plankton databases (this analysis) is given in Table S2.

### Spatial and temporal patterns

**Figure 3:**
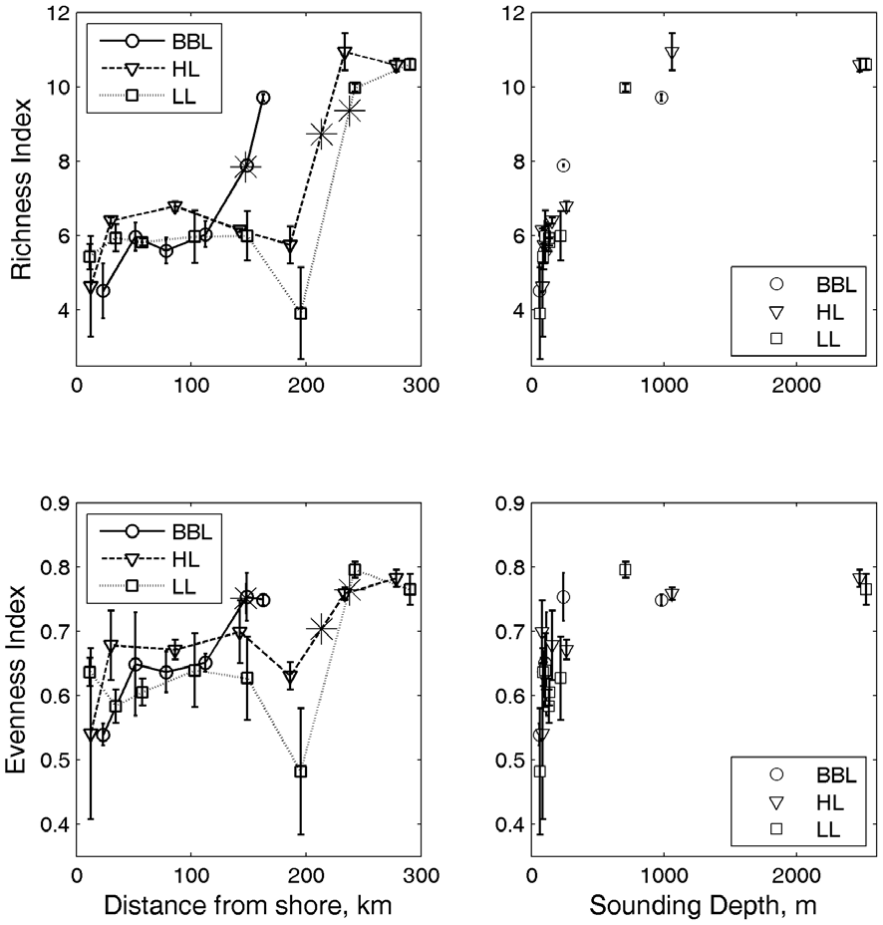
Relationships between copepod species richness and evenness and distance from shore and depth on the Scotian Shelf. Data on adult copepods were collected by the Atlantic Zone Monitoring Program during spring and fall cruises on the Scotian Shelf between 1999 and 2008. Richness index based on rarefaction to 50 individuals.Bars indicate standard error. The * symbol indicates the location of the shelf break, defined as the 200 m isobath, along each transect. BBL – Brown's Bank Line; HL – Halifax Line; LL – Louisbourg Line.

On the coastal shelf of the western Gulf of Maine, adult copepod diversity along the WBL transect from 10 to 75 km off shore tended to be higher near the center of the transect, but diversity was not significantly different among stations (two-way ANOVA with station and season as factors; *p_station_*  = 0.155, *p_season_* <0.001, p_station*season_  = 0.582; Figure 2; data collection methods described in [40]). Differences in copepod evenness and species richness among coastal shelf stations were also not significant. Abundant copepod species typical of neritic or shelf communities were found at all stations along the transect at some time during the time series, reflecting mixing of the neritic and shelf communities on the coastal shelf. In contrast, the diversity of higher-level taxonomic groups in the same region declined with distance from shore between about 10 and 75 km, reflecting the higher diversity of meroplankton in the nearshore environment (two-way ANOVA with station and season as factors; *p_station_* <0.001, *p_season_*  = 0.003, p_station*season_  = 0.001; Figure 2; data collection methods described in [40]). On cross-shelf transects on the Scotian Shelf, copepod species richness and evenness were higher in the slope waters than on the shelf, due to mixing of the diverse off-shelf communities and the continental shelf community (Figure 3). Inshore of the shelf break, richness and evenness were relatively low, but both were lowest on offshore banks and in shallow inshore waters, reflecting a non-linear, increasing trend of richness and evenness with bottom depth (Figure 3). We are not aware of any quantitative studies examining cross-shelf trends in species richness inshore of 10 km in the Gulf of Maine or Scotian Shelf region.

**Figure 4:**
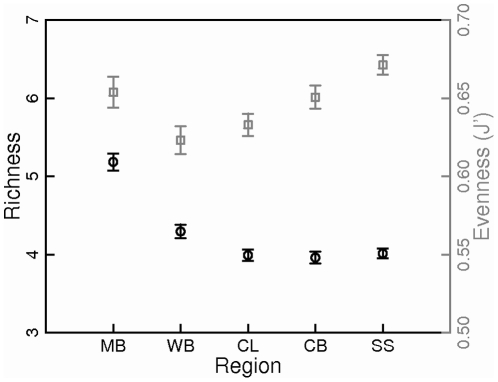
Regional variation in copepod species richness and evenness in the Gulf of Maine. Richness and evenness were based on standardized Continuous Plankton Recorder samples. Black circles - richness; gray squares - evenness. MB - Massachusetts Bay; WB - Wilkinson Basin; CL - Cashes Ledge; CB - Crowell Basin; SS - Scotian Shelf Inflow. Bars indicate standard error. N = 477, 666, 1024, 851, and 1172, respectively.

**Figure 5:**
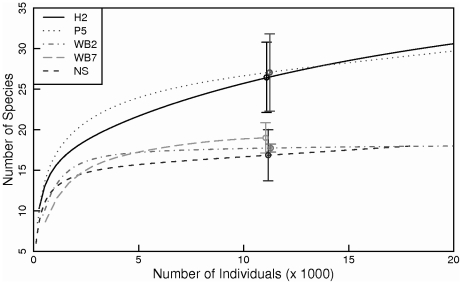
Species accumulation curves for adult copepods at time series stations in the Gulf of Maine. H2 – Halifax Line station 2; NS – New Scantum; P5 – Prince-5; WB2 and WB7; Wilkinson Basin Line stations 2 and 7, respectively. 95% confidence intervals were estimated for each station at the highest common number of individuals, N = 11,168.

At the regional scales measured by the CPR transect, average, per-sample copepod species richness (across months and years) was highest in Massachusetts Bay (Figure 4), due to the persistent presence of both neritic and shelf species in this region. Richness was also somewhat higher in the western Gulf of Maine (Wilkinson Basin region) than in the central and eastern regions (Figure 4). In contrast, copepod species accumulation curves based on data from coastal shelf stations in the western Gulf of Maine, Bay of Fundy, and central Scotian Shelf indicate that copepod diversity was lower in the western Gulf than in the Bay of Fundy and Scotian Shelf (Figure 5). While this discrepancy may be due to differences in sampling depth or location between the CPR and fixed-station sampling programs, we believe that the higher species richness observed in species accumulation curves at the eastern stations is due to the transient appearance of offshore or cold-water species on the Scotian Shelf and in the Bay of Fundy. The contribution of these rare species is not captured in averaged sample-based species richness estimates, which are more representative of the dominant community at a particular location and time period. The larger overall numbers of copepod species observed in species accumulation curves compared to averaged sample-based species richness estimates reflect both the influence of sample size on species richness estimates as well as temporal community variability at sub-seasonal to interannual timescales. Copepod species evenness, based on CPR samples, was high in both the eastern Gulf of Maine (Scotian Shelf and Crowell Basin regions) and in Massachusetts Bay, while it was lower in the central and western Gulf, suggesting stronger dominance of a few species in the central and western Gulf of Maine (Figure 4).

**Figure 6:**
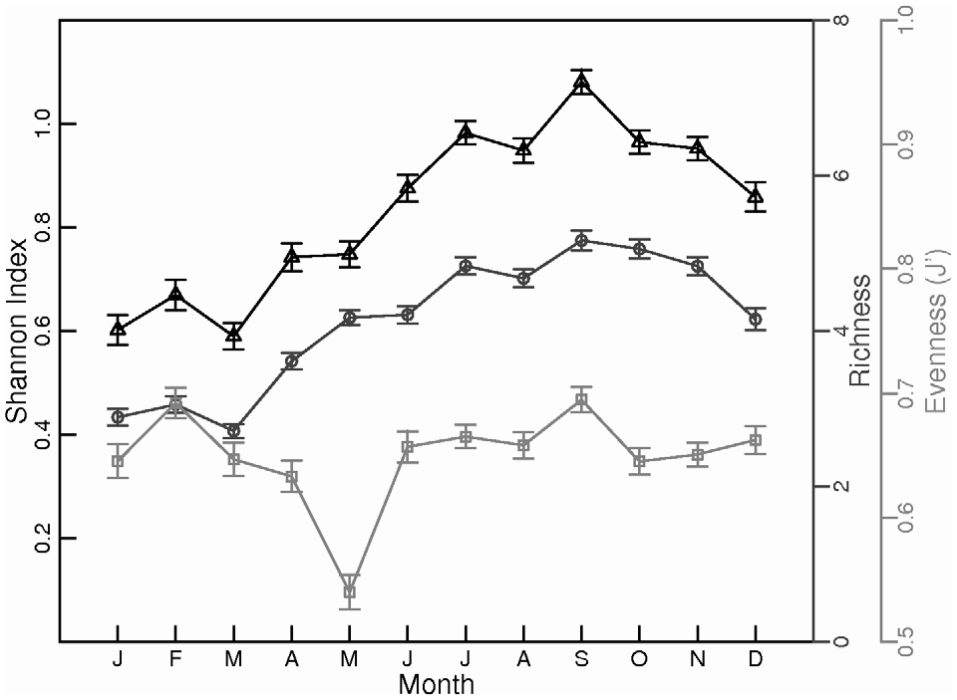
Seasonal variation in copepod species richness, evenness, and diversity in the Gulf of Maine. Richness (circles), evenness (squares), and diversity (triangles) were based on standardized Continuous Plankton Recorder samples. Bars indicate standard error. Sample size ranges from 289 to 422.

Average copepod diversity across years and regions, estimated from CPR survey data using the Shannon (H') index, exhibits an annual cycle, with the maximum diversity in the summer and early fall in the Gulf of Maine (Figure 6). High summer and fall copepod diversity was driven mainly by high richness during these seasons (Figure 6). Manning & Bucklin [40] observed a similar annual cycle in copepod species richness, based on net-collected samples in the western Gulf. Evenness did not exhibit a strong seasonal cycle in either the CPR surveys or in Manning & Bucklin's study [40], but local minima in evenness were sometimes observed during blooms of dominant species such as *Calanus finmarchicus*, *Temora longicornis,* and *Centropages typicus* (Figure 6; [40]). Seasonal variability patterns in diversity indices were not consistent across the GoMA. At the Prince-5 station near the western shore of the mouth of the Bay of Fundy, the Shannon diversity index for copepods was also highest in the late summer and early fall, but this pattern was driven by seasonal variation in evenness rather than richness, which does not have a strong seasonal cycle at this site (C. Johnson, E. Head, A. Curtis, personal communication). At the Halifax-2 station on the central Scotian Shelf, the Shannon diversity index for copepods was high from spring through early fall, influenced by seasonal variation in both species richness and evenness (C. Johnson, E. Head, A. Curtis, personal communication).

**Figure 7:**
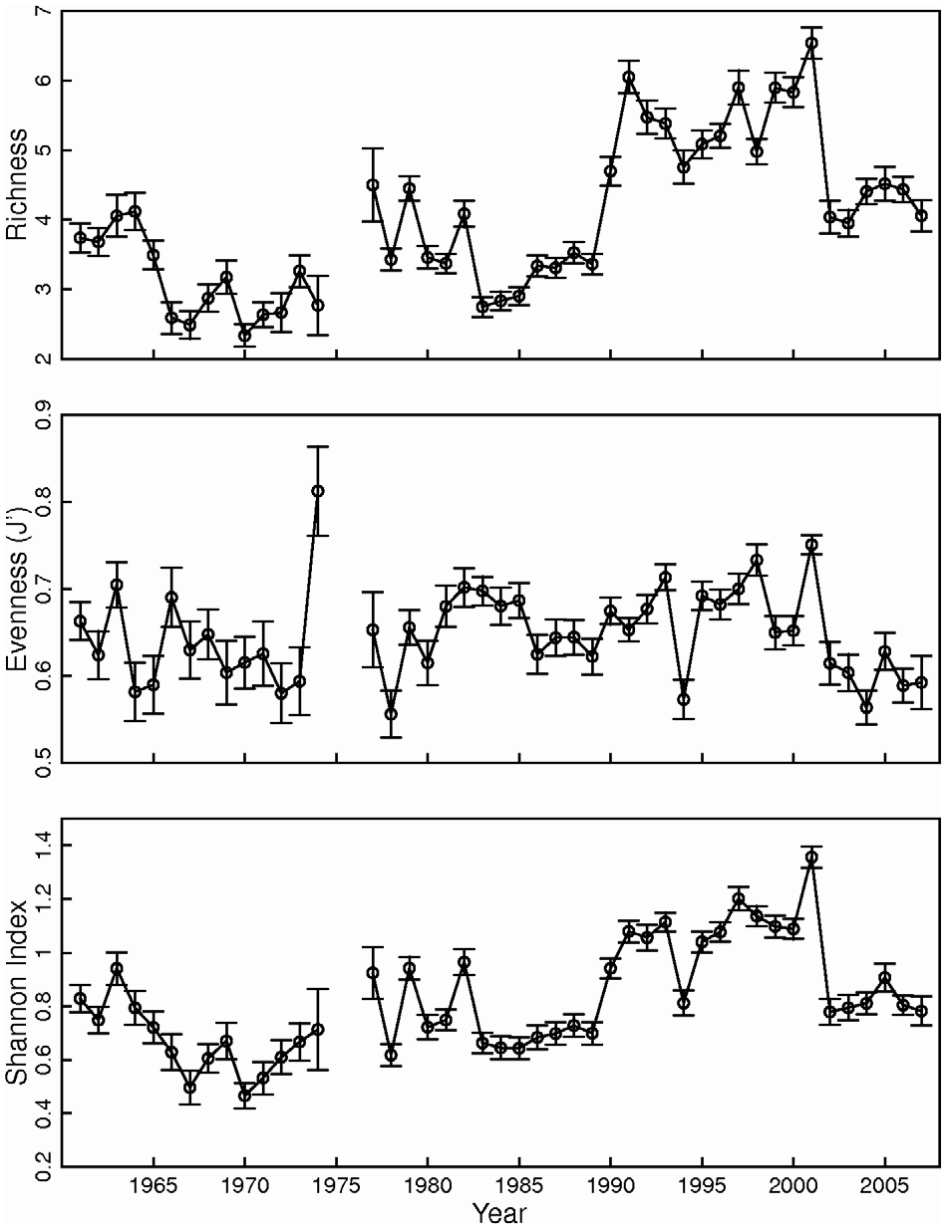
Interannual variation in copepod species richness, evenness, and diversity in the Gulf of Maine. Richness, evenness, and diversity were based on standardized Continuous Plankton Recorder samples. Bars indicate standard error. Mean sample size  = 94, s.d. = 38.

There were distinct interannual patterns in copepod diversity, driven more by species richness than evenness, over the 40+ years of CPR data (Figure 7). The Shannon diversity index for copepods decreased through the 1960's and then slowly increased in the 1970's and early 1980's. There was a marked jump in copepod diversity in 1990 that lasted through 2001, when values returned to pre-1990's levels. In contrast to the temporal and spatial patterns observed in zooplankton abundance, changes in species diversity, in particular species richness, documented in the CPR data were greatest on interannual scales, intermediate on seasonal scales, and smallest across regions. Thus, zooplankton diversity may be a more sensitive indicator of ecosystem response to interannual climate variation than zooplankton abundance.

### Functional groups

**Figure 8:**
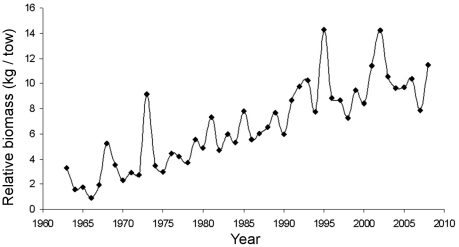
Interannual variation in the relative biomass of the major small pelagic fishes in the Gulf of Maine region. Adapted from [32].

Pelagic species can be organized in a variety of ways, including taxonomically-, functionally- and energetically-based groupings. Groupings such as trophic guilds and habitat assemblages that have been used to classify and categorize species groupings address different aspects of diversity. Pelagic functional groups make different relative contributions to the flow of energy and biomass in the GoMA food web (Table 2). Biomass is concentrated at lower trophic levels and declines at higher trophic levels, similar to patterns of production for functional groups [6] [16]. The pelagic community in the GoMA can be influenced by the specific attributes of the species comprising each functional group, and the biomass of individual species relative to the total biomass in these functional groups has changed over time, for both invertebrate and fish groups. Dramatic shifts in key members of functional groups have also been observed, for example an increase in the relative abundance of ctenophores, as estimated through fish diet analysis [41]. Similarly, the biomass of various pelagic fishes has changed over time in response to both fishing pressure and broad-scale environmental conditions [42]. The entire group of pelagics has increased in abundance and biomass since the mid-1960s (Figure 8; *cf.* [43] [7]). This pattern is stronger for certain individual species, such as Atlantic herring or Atlantic mackerel, but in aggregate there is group level compensation by other species of small pelagics. Group-level compensation is most marked in the piscivorous, benthivorous, and amphipod-shrimp-feeding guilds, in which the abundance trajectories of individual species have fluctuated, but the relative constancy of the major functional guilds have remained relatively constant [3].

**Table 2:** Major functional groups of pelagic species in the Gulf of Maine and standing stock biomass estimates.

Group	Biomass (t km^-2^)
Phytoplankton- Primary Producers	20.11
Bacteria	3.45
Microzooplankton	3.16
Small copepods	9.88
Large Copepods	34.85
Gelatinous Zooplankton	11.0
Micronekton	8.36
Mesopelagics	3.66E-05
Shrimp et al.	0.169
Larval-juvenile fish- all	0.258
Small Pelagics- commercial	4.54
Small Pelagics- other	1.06
Small Pelagics- squid	0.135
Small Pelagics- anadromous	0.0772

Adapted from [16].

## Discussion

### Drivers of Gulf of Maine pelagic biodiversity

In the open ocean, away from continental shelves, local factors are relatively unimportant as drivers of zooplankton and pelagic nekton biodiversity, but in shelf seas, where the physical environment is more strongly influenced by the coast and the bottom, local factors such as habitat, predation, and advection have a stronger influence on diversity [44]. The GoMA is a shelf sea, and the spatial zooplankton diversity patterns described in the present study primarily reflect the underlying spatial distribution of the neritic, continental shelf, and offshore communities [30] [45] [46] [47] [48] [49] [50] [51] [52] [53].

Low salinities and variable conditions found in nearshore bays and estuaries provide a harsh environment for zooplankton and reduce diversity in these nearshore areas [54]. Benthic-pelagic interactions also influence nearshore zooplankton communities through increased the risk of predation by benthic planktivores and through utilization of benthic habitat during dormant periods. Neritic zooplankton communities of the GoMA include many species that can produce “resting eggs”, dormant embryos that can survive through unfavorable periods in a refractory state in the sediment. These species include the copepods *Acartia longiremis, A. hudsonica, A. tonsa,* and *Eurytemora herdmani,* and *Tortanus discaudatus,* and the cladocerans *Evadne nordmanni* and *Podon* species [55] [56] [57] [58]. These and other dominant nearshore copepod species such as *Pseudodiaptomus pelagicus* are tolerant of low salinities [54]. The neritic community also includes a rich assemblage of meroplankton, the pelagic early life stages of benthic organisms, including barnacles, bivalve and gastropod mollusks, decapod crustaceans, echinoderms, worms, and bryozoans [45] [48] [59] [60]. Mysids, which utilize both benthic and pelagic habitats as adults, are abundant members of GoMA neritic communities [61] [62].

In the central Gulf of Maine and on the Scotian Shelf, the copepods Calanus finmarchicus, Centropages typicus, Metridia lucens, Microcalanus pusillus, Microsetella norvegica, Oithona similis, Paracalanus parvus, and Pseudocalanus species are dominant species, and non-copepod taxa such as chaetognaths, amphipods, euphausiids, ctenophores, and pteropods are also important members of the community [45] [46] [63] [56] [64]. Communities on the coastal shelf outside of bays and estuaries include neritic and central Gulf/shelf species as well as broadly distributed species such as the shallow water copepods Temora longicornis and Centropages hamatus, which are common both close to the coast and on offshore banks [49] [65] [66] [67]. The small, shelf copepod species, Oithona similis and Paracalanus parvus, are also numerical dominants in Massachusetts Bay in the western Gulf of Maine [52]. The predominance of the large copepod Calanus finmarchicus is notable in both the coastal shelf and offshore metazoan zooplankton communities, particularly in late winter and spring [45] [46] [30]. While many zooplankton species are common to both the central Gulf of Maine and western Scotian Shelf [64], the western Scotian Shelf community is also influenced by cold-water species advected from the Gulf of Saint Lawrence, such as Calanus hyperboreus and C. glacialis [68], and by warm-water taxa such as Mecynocera clausi and Clausocalanus species, advected from offshore waters [69] [64]. These cold- and warm-water species are not abundant in the central and western Gulf of Maine, but they are more often observed in the eastern Gulf of Maine and Bay of Fundy.

At a local scale, neritic communities tend to be less diverse than oceanic communities [70]. Observations of copepod diversity in the Bay of Fundy were consistent with this trend: zooplankton diversity was lower in the inner Bay of Fundy than in the outer Bay [61]. In the western Gulf of Maine, however, cross-shelf diversity patterns of both copepods and higher-level taxonomic groups did not exhibit this pattern. For copepods, the absence of significant cross-shelf trends in Shannon diversity, richness, and evenness along the western Gulf transect reflected the limited spatial scale of the transect, lack of sampling closer than 10 km from shore, and the broad zone of mixing between the near-shore neritic and central Gulf copepod communities. Central Gulf copepods were found even at the inner-most station, and near-shore species were found at the outer-most station. The contrasting observation of a decline in the diversity of higher-level groups with distance from shore reflects the greater prevalence of benthic taxa with planktonic larval stages at stations near the coast. At a broader scale, the higher copepod species richness observed on CPR surveys in Massachusetts Bay compared to the deep-water central and eastern Gulf also reflects mixing of the neritic and central Gulf communities on the western Gulf of Maine coastal shelf.

As indicated in the western Gulf of Maine, advection can produce zones of mixing among communities on the coastal shelf. Advection can alter species diversity in the nearshore, for example through transport of shelf species into estuaries via deep, onshore flow of offshore water [71]. The potential for advection to dramatically alter local species diversity on continental shelves has been documented in the coastal Northeast Pacific [72] and in the coastal Northeast Atlantic, where the well documented Russell cycle (e.g. [73]) in the western English Channel is likely the consequence of shifts in the circulation of the basin scale subpolar gyre [74]. While the dominant circulation pattern in the GoMA is not conducive to such large-scale biogeographic boundary shifts, new species may nonetheless be introduced either from surface inflow from the Scotian Shelf or from inflow of slope water in the Northeast Channel. Warming temperatures, including shifts in annual maxima and minima and season lengths, could allow the expansion of some species whose populations presently live south of Cape Cod or occupy small refuges within the GoMA. Shifts in diversity can be a sensitive indicator of system change, especially if knowledge of the life history enables a mechanistic (e.g., oceanographic, physiological, ecological) explanation.

On the Scotian Shelf, copepod species richness and evenness increased non-linearly with bottom depth. In an ocean-basin context, this region is influenced by its geographic location close to 40°N latitude, where a shift in zooplankton evenness and taxonomic distinctness shift was previously noted [75] [76]. The observed relationship between diversity and bottom depth on the Scotian Shelf reflects a greater contribution of the comparatively stable, more southern, offshore zooplankton community in slope waters and deep shelf basins than on shelf banks. The shelf community in this region is similar to northern oceanic communities in its strong seasonality, high variability, and lower diversity. The influence of immigration from the offshore community onto the shelf is manifested in higher copepod diversity at stations sampled year-round on the Scotian Shelf and in the Bay of Fundy, compared to stations in the western Gulf which have less influence from the offshore environment.

At a latitudinal, basin scale, zooplankton diversity patterns are strongly correlated with annually averaged sea surface temperature and to a lesser extent, salinity, and negatively correlated with average sea surface chlorophyll [77]. The drivers of these large scale diversity trends have been hypothesized to involve a suite of mechanisms directly or indirectly linking higher energy to diversity, for example through higher overall abundance and rates of speciation at higher temperatures [77] [78]. Alternatively, the seasonality of food and temperature cycles at higher latitudes is hypothesized to reduce species richness relative to the subtropics and tropics, where greater environmental stability allows greater vertical niche partitioning and specialization, including a greater proportion of carnivorous species [76] [79] [80] [81]. In the higher latitudes, food scarcity during part of the year forces species to be either generalist feeders, such as species in the genus *Oithona*, or overwintering lipid storers, such as species in the genus *Calanus*. The consequence of this seasonality is limitation in realizable niches. It is interesting to note, however, that seasonality can promote alternation of dominant congeners of calanoid copepods, and that, while in estuaries cyclopoid species richness is higher at lower latitudes, calanoid species richness does not show a significant latitudinal gradient among estuaries along the east coast of North America [82].

This study and others have described decadal-scale shifts in copepod diversity and in the relative abundance of the dominant copepod species, notably toward order of magnitude higher abundances of smaller taxa (*Pseudocalanus, Oithona, Centropages*) and lower abundance of large, late stage *Calanus* in the Gulf of Maine and on Georges Bank in the 1990's [47] [83] [84]. Pershing et al. [47] and Kane [83] linked the zooplankton community change of the 1990s to salinity anomalies that originate at high latitudes [85], suggesting that changes in GoMA pelagic diversity are forced by external processes [86]. The increased abundance of small copepod species is hypothesized to be driven either by increased fall stratification, leading to more intense and longer duration fall phytoplankton productivity [47] [86], or to increased influx of zooplankton from the Scotian Shelf [83]. Predation from forage fish, in particular herring that increased dramatically during the same time period, also may have contributed to a reduction in the abundance of late, lipid-rich stages of *Calanus finmarchicus* in the western Gulf of Maine [86] and eastern Scotian Shelf [87]; however studies of fish stomach-contents from multiple small pelagic species have not confirmed this hypothesis for Georges Bank or the Gulf of Maine [88](J. Link, personal communication).

### The “Underknown” species and groups

While long-term zooplankton monitoring efforts in the GoMA are adequate to identify spatial and annual variability patterns in the offshore mesozooplankton community, especially the copepods, many other zooplankton and pelagic nekton groups have not been sampled adequately. These “underknown” species and groups are likely to have important roles in the ecosystem that only become evident with better assessments of their abundance and interactions. Many of the now commercially dominant and most valuable species, such as monkfish, were effectively ignored even 30 years ago [89]. Studying these “underknowns” will provide better knowledge of those components of the food web that could be the driving forces and/or major target species in future fisheries. Species that we suspect to be major drivers of ecological functioning in the GoMA ecosystem (e.g., gelatinous zooplankton, euphausiids, mesopelagic fishes) are all understudied, yet remain critical elements of the food web in this region [6] [15] [16] and will require further attention for successful implementation of ecosystem-based management in the GoMA.

#### Invertebrate meroplankton

The larvae of a variety of benthic organisms including crabs, barnacles, bivalves, echinoderms, and bryozoans contribute to zooplankton diversity in the GoMA, especially in bays, estuaries, and the near-shore ocean [45] [48] [30]. Many benthic organisms produce planktonic eggs or larvae in short, intense pulses, and thus meroplanktonic taxa are abundant or even dominant members of the zooplankton community for brief periods [90]. Current GoMA zooplankton monitoring efforts are likely undersampling the meroplankton due to both the transience of meroplankton production and an emphasis on sampling primarily outside of the near-shore waters where meroplankton are most abundant. Nevertheless, these species are important prey for larval, juvenile, and adult fish in estuaries and coastal waters [91], and their seasonal and interannual dynamics may influence fish recruitment variability and zooplankton community dynamics. Nearshore meroplankton may be more susceptible to human impacts than offshore zooplankton and ichthyoplankton, due to land-based sources of pollution and alteration of the shoreline and nearshore habitat where the benthic phases of these taxa reside. Some meroplankton species are commercially important, for example the soft-shell clam (*Mya arenaria*), hard-shell clam (*Mercenaria mercenaria*), northern shrimp (*Pandalus borealis*), lobster (*Homarus americanus*), sea scallop (*Placopecten magellanicus*), and bay scallop (*Argopecten irradians*); however, with few exceptions (e.g., [92]), the spatial and temporal patterns of their larvae are not well described or understood.

#### Gelatinous Zooplankton

Gelatinous zooplankton, principally hydromedusae, scyphomedusae, siphonophores and ctenophores, have long been recognized as characteristic components of the GoMA plankton [45]. Cnidarians and ctenophores are predators, mainly on crustacean zooplankton, fish eggs and larvae, juvenile and adult fish (for the larger scyphomedusae) and other gelatinous animals. Pelagic tunicates (salps, doliolids, pyrosomes, appendicularians) are filter-feeding omnivores. Biomass and probably predation impact can be very high periodically for species such as *Pleurobrachia*, *Bolinopsis*, *Nanomia* or *Clytia* hydroids that have rapid rates of reproduction and growth (e.g. [93]). Aggregations of gelatinous zooplankton can also impact fishing gear; for example, siphonophores were blamed for the ‘lipo’ phenomenon which fouled commercial fishing nets in the 1970's [94]. Gelatinous zooplankton populations are difficult or nearly impossible to quantify with conventional sampling because they are often patchy, ephemeral and too fragile to survive net sampling [95] [96]. However, there are enough observations by divers or submersibles to indicate that these organisms can be extremely abundant. Bigelow [45] listed about 20 species, most of which have also been reported in later years from sampling programs such as the U.S. GLOBEC Georges Bank/Northwest Atlantic program. Recent submersible-based investigations in the Gulf basins and marginal canyons and additional sampling efforts in the future will likely find new species. Based on stomach samples of the spiny dogfish *Squalus acanthias,* Link and Ford [41] suggested that there have been dramatic increases in ctenophores in the northeast U.S. shelf ecosystems, potentially changing predation pressure on pelagic communities. There is a need to develop new approaches to observe changes in abundance and distribution of gelatinous zooplankton in the GoMA and to describe the strength of their trophic interactions in order to quantify their effects on the ecosystem.

#### Mesopelagic Fishes

System-wide models of upper trophic levels in the GoMA suggest that an important component of fish biomass remains unquantified [15] [16]. This “missing biomass” is believed to consist of myctophids and other mesopelagic micronekton that may be abundant in deep water along the southern flank of Georges Bank and in the northeast Channel. Low estimates of biomass for this group (Table 2) may result from inadequate sampling. It is likely that some of this biomass migrates from the continental slope, and some may represent a vertically migrating resident stock in some regions of the GoMA [97] [98] [99] [100]. Many of the offshore, shelf-break and shelf-slope fish communities are also poorly understood. Although species lists are now being compiled (e.g. [98] [99] see also [101]), the fullness of those lists, let alone the functioning, vital rates, and interactions of those species, remains essentially unknown [102].

The location and composition of the missing biomass and the role that it ultimately plays in the ecosystem are presently unknown. Estimates are needed of the biomass, production, and unique deep water life histories of mesopelagic fishes along the continental shelf slope, associated canyons/sea mounts, and basins within the Gulf of Maine, as well as the rate of flux of mesopelagics onto and off of Georges Bank. It is possible that mesopelagic fishes serve as both prey for and competitors with juvenile groundfish while at the same time preying on larval groundfish as well as lipid-rich *Calanus* life stages. The larval and adult stages of the mesopelagics are strongly associated with major oceanographic fronts and the edge of the Gulf Stream (e.g., [97] [103] [104] [105]), and thus the positions of these features may have implications for groundfish recruitment. Estimates of the rate of consumption by highly migratory megafauna such as billfish, tunas and marine mammals would provide insight into the importance of mesopelagics as a source of food for megafauna, particularly along the shelf slope front, with implications for their recruitment and patterns of migration. Establishing a clear link to better known megafauna would help to elucidate the role of the mesopelagics in the regional food web.

#### Euphausiids

Euphausiids, notably the carnivorous *Meganyctiphanes norvegica*, are important constituents in the diet of upper level carnivores in the GoMA. Euphausiids may also be strong predators on planktonic copepods. Eight species of euphausiids have been reported in the GoMA, six from the interior of the Gulf (*Thysanopoda acutifrons*, several *Thysanoess*a spp., and *M. norvegica*; all listed in GoMRMS) and two from the slope (*Nematoscelis megalops* and *Euphausia krohni* [45] (NOAA Northeast Benthic Database). Sixteen additional species were identified from the BioChem database, mostly at the deepest stations on the Halifax Line (Table S2 and Fig. 3). The slope species may on occasion be transported into the Gulf. *Thysanoessa longicaudata*, *T. inermis* and the large *M. norvegica* are broadly distributed, and the latter two are abundant and probably have significant roles as planktivores and as prey for fish and other organisms. For example, euphausiids, notably *M. norvegica*, were found to constitute approx. 30% on average of the biomass in the diet of Atlantic herring in coastal waters of the GoMA. All three have northern affinities, and *M. norvegica* is noted for forming large surface swarms during warm months of the year, particularly in the northern Gulf. The swarms attract vigorous feeding by herring and whales [45] [106] [107]. *M. norvegica* are large (adults are >2 cm body length), swim rapidly and avoid collection by traditional net sampling devices. Consequently, their distribution, seasonal abundance and population dynamics in the GoMA are poorly known. The role of euphausiids, especially *T. inermis* and *M. norvegica*, in trophic processes is a significant gap in our understanding of the Gulf. There is a need for survey approaches, such as sampling by large, strobe light equipped nets or acoustic methods [108], as well as ecological studies to better assess their distribution and role in the GoMA ecosystem.

#### Mysids

There is growing recognition of the important role of the Mysidacea (commonly known as oppossum shrimp) in shallow coastal ecosystems of mid-latitude continental shelves. Although frequently observed in high abundances, mysids are nevertheless likely to be underrepresented in marine food web models due to sampling challenges and a paucity of research focused on mysid ecology [62]. The mysids *Neomysis americana, Erythrops erythrophthalma*, *Americanysis bigelowi* and *Mysis mixta* have been observed in the GoMA[109] [110]. *N. americana* is the most common, occurring in shelf habitats from 200 m deep to estuaries. This species is known to undertake diel migrations from the bottom into the water column, particularly during summer and fall, and it may also undertake seasonal, horizontal migrations from nearshore to offshore during winter [62]. Because of their omnivorous benthic and pelagic existence, mysids are likely predators on a wide range of benthic and pelagic species, and they also serve as prey for both demersal and pelagic fishes, connecting benthic to pelagic and nearshore to offshore food webs and likely enhancing stability in the GoMA ecosystem.

#### Squid

The loliginid (long-finned squid), *Loligo pealeii*, and the ommastrephid (short-finned squid), *Illex illecebrosus*, are the most commonly reported species of pelagic squid in the GoMA [111]. Although these species are sympatric, the distribution of *L. pealeii*, which is known to spawn in shallow waters of the mid Atlantic bight, is typically more neritic than the more migratory and oceanic *I. illecebrosus*, which is reported to spawn mainly in winter off the continental shelf south of Cape Hatteras [112]. As consumers of juvenile fish, euphausids, mysids and other zooplankton [113] [114], and as prey for several top predators in the GoMA [115], pelagic squid may play an important role in the GoMA ecosystem. Both species have a relatively short (1-2 yr) life span and are highly variable in abundance interannually[111] [112]. *L. pealeii* predation and possibly competition is hypothesized to have a primary influence on marine fish recruitment in northwest Atlantic coastal waters in years when it is abundant [116]. The strength of these food web interactions and the interannual and longer term variation in spatial distribution and abundance levels of pelagic squid in the GoMA are not well studied. The potential influence of squid on food web dynamics and the relative abundance of its prey taxa require closer attention.

### Food web interaction strength, trophic linkages and ecosystem shifts

Identifying and quantifying trophic interactions among zooplankton, pelagic nekton, and other marine species is critical to understanding ecosystem structure and function. The GoMA food web is complex, even though the zooplankton community is dominated by relatively few species when compared to other groups [117]. Once species and population diversity patterns have been described, elucidating functional diversity requires an understanding of how species are connected and interact ecologically. Translating linkages (*sensu* [117]) into energy flows [6] [15] [16] and interaction strengths remains an important challenge, particularly for species whose roles in the ecosystem are still unknown, or if the dominance structure of communities changes. Without reliable estimates of trophic linkages or interaction strengths, many models that are used to support ecosystem-based management will not be adequately parameterized.

One approach to understanding climate and predation impacts on constituent species and potential implications for higher trophic levels is to model particular compartments in local food web structure (e.g. [118]). For example, changes in phytoplankton composition and bloom timing may lead to shifts in the seasonal timing of dominant copepod species such as *Calanus finmarchicus*, with consequences for herring foraging [119] and subsequently for higher trophic levels. Analysis of zooplankton community structure in the northeastern Atlantic shows biogeographic and ecosystem shifts associated with the northward movement of the 9–10°C SST isotherm [120] [121], including species replacements [121], changes in abundance of holozooplankton and meroplankton [122] [123], phenological shifts and trophic mismatches [124]. Notable is the evidence for replacement of the key structural, subarctic planktonic copepod, *Calanus finmarchicus*, by its warm water congener, *C. helgolandicus*. This change in *Calanus* spp., with their differences in life histories [125], is implicated in the observed long-term changes in cod recruitment in the North Sea [126].

Climate impacts on water column temperature and circulation are different in the northwest Atlantic than in the northeast Atlantic. The global trend of rising SST may be offset in the northwest Atlantic region by greater transport of colder, Labrador shelf water into the Gulf of Maine due to the freshening of seawater in the Arctic Ocean and Labrador Sea [86]. Nevertheless, the potential for a biogeographic shift in distribution of *C. finmarchicus* is of concern because of its key structural role in the GoMA ecosystem. In addition to being the biomass dominant mesozooplankton species in the deep Gulf of Maine and often along the coastal shelf, *C. finmarchicus* has the capacity to store high quantities (>60% of its total body mass) of energy-rich lipids, which are used to sustain pre-adult stage individuals during the overwintering diapause period and prepare for subsequent molting and reproduction in late winter. Examination of the diapause and population dynamics of *C. finmarchicus* [127] [128] (F. Maps, personal communication) indicates that the ambient overwintering temperature for *C. finmarchicus* in the Gulf of Maine is on the high end of its biographic range, such that water column warming, for example due to changes in the input of warm slope water to the deep gulf or changes in winter convection and deep-water cooling, would impact timing of entry into and emergence from diapause. Possible consequences of this change are mismatches with the primary production cycle, with the seasonal presence of larval fish that may rely on the early developmental stages of *Calanus* as a primary food source, and with seasonal feeding cycles of planktivorous fish such as herring and mackerel that prey on older copepodid stages. At some point, the conditions of overwintering temperature and the timing and magnitude of food availability may combine to make populations of *C. finmarchicus* unsustainable in the Gulf of Maine, at which point a biogeographic shift would occur.

The extent to which other zooplankton species would fulfill the role of *C. finmarchicus* is currently unknown, but there is no large, resident, non-*Calanus* species with equivalent lipid content in summer. Substantial reduction in *C. finmarchicus* abundance in the Gulf of Maine could trigger a regional ecosystem shift, because it is the most prominent, lipid-rich energy source for planktivorous species such as herring, mackerel, sand lance, northern right whales, and phalaropes, and an indirect energy source for large pelagics such as bluefin tuna that feed on planktivorous forage nekton [120] [121]. The question of whether *Calanus* is a keystone species with strong interactions or one of 10–20 copepods species with weak interactions is critical to understanding potential changes in the GoMA ecosystem structure and function. To date there is no evidence of substantial decreases in *C*. *finmarchicus* abundance in the Gulf of Maine, but CPR data suggest a shift to greater numerical dominance of the small copepod group *Para-* and *Pseudocalanus* compared to *Calanus finmarchicus*, in the northwest Atlantic between the 1990s and the 1960s/1970s [11].

### Population studies and metapopulation analysis

The population structure of zooplankton and pelagic nekton in the GoMA is an important consideration for the management of living marine resources. Species that are present in distinct sub-populations must be considered in terms of metapopulations - a group of several local populations linked by immigration and emigration. Here, a sub-population is one in which the life cycle can be completed within a geographically discrete region. Zooplankton such as *Acartia tonsa* are restricted to individual estuaries with little exchange between them and may form genetically distinct sub-populations [129]. The copepod *Calanus finmarchicus* requires a region where it can undergo diapause in order to complete its life cycle - in shallow regions such as Georges Bank where it may be seasonally very abundant, it is an expatriate and not a separate self-sustaining population [130]. Thus, in the GoMA *C. finmarchicus* could be considered as connected to other population centers in the Western North Atlantic including the Slope Water, Scotian Shelf, and the lower St Lawrence Estuary/Gulf of St Lawrence. For many species of zooplankton, however, we do not know enough about their distribution or their life cycle within different regions to evaluate the spatial structure of their metapopulations. For pelagic fish, which have a bi-phasic life history (planktonic larval stage; adult free swimming and possibly wide ranging stage), the region to which the adults return to spawn provides a separation of sub-populations [131]. Connectivity among sub-populations needs to be determined using techniques such as tagging and otolith microchemistry for adult fish, or population genetics to measure gene flow between populations for larval fish or zooplankton. Individual-based transport modeling can also contribute to understanding connectivity (e.g. [132] [133]). In addition, the influence of environmental conditions and habitat, including climatic and anthropogenic changes, on phenotypic expression and genetic composition, including whether sub-populations show different geographic responses, needs to be evaluated.

### Observing change in biodiversity

Based on our current knowledge of the relationships between zooplankton and pelagic nekton communities and the environment, we expect that changes in large scale forcing and water-mass contributions could cause ecologically significant shifts in both species diversity and the ecosystem functions performed by the zooplankton and pelagic nekton communities. Zooplankton community responses to climate change are likely to manifest themselves as biogeographic shifts and changes in seasonal timing as well as species introductions. The possibility of a relatively sudden change to a different ecosystem state, perhaps even driven by zooplankton community change, cannot be ruled out (e.g. [134]). While fish diversity in the GoMA has not notably changed [32] [135], at least in terms of species richness, it remains to be seen how diversity and ecosystem processes will change as climate forcing and fishing mortality continue to influence the zooplankton and fish assemblages in this ecosystem [42].

The high taxonomic resolution and extensive sampling effort of the current and past zooplankton monitoring programs make them suitable for use in identifying mesozooplankton diversity and its variability. Nevertheless, establishing a comprehensive zooplankton diversity baseline is challenging due to differences in gear type, mesh size, sampling depth and distribution, and taxonomic resolution among plankton monitoring programs. Monitoring efforts by the EcoMon and AZMP monitoring programs are adequate to resolve spatial and interannual variability patterns of dominant mesozooplankton in the offshore waters of the Gulf and Scotian Shelf, but additional observations would be needed to detect changes in coastal and estuarine components of the GoMA ecosystem and at the upstream and offshore biogeographic boundaries. At present, only the AZMP station on the Scotian Shelf is adequate to detect changes in zooplankton phenology at sub-monthly scales. Detection of seasonal changes on the scale of 2–4 weeks in the GoMA would require a carefully-selected set of high frequency time series stations comparable to the Scotian Shelf station. Additional sampling with multiple gear types and mesh sizes would be necessary to observe biodiversity across the full taxonomic and size range of zooplankton and pelagic nekton, including “underknown” species and groups such as those discussed above. While advanced technologies for measuring zooplankton, such as the optical plankton counter (OPC), video plankton recorder (VPR), and acoustics provide inadequate taxonomic resolution for monitoring biodiversity of mesozooplankton, acoustics and video recording may be helpful in assessing abundance changes of some of the under-sampled groups. Broader use of genetic tools would benefit identification of certain taxa of interest, such as meroplankton, as well as spatial genetic structure of populations.

Observations of changes in diversity would be enhanced by the application of alternative approaches to characterizing diversity and the use of multivariate ordination methods to identify and visualize interactions among plankton, fish and environmental variables. The species richness, diversity, and evenness indices used here are influenced by sampling intensity (number of samples collected at a site and the number of individuals counted per sample) [136] [137]. Low species evenness (e.g., nearshore and shelf stations in Figure 3), exacerbates the influence of sample size ([138] cited in [139]). Rarefaction can be used to standardize the effects of sampling effort on observed species richness [140]. Simulations based on a range of well-sampled zooplankton communities should be used when designing monitoring programs to estimate the minimum sampling effort for reliable species richness and diversity comparisons. Alternative diversity metrics (e.g., the Simpson diversity index, which provides a direct measure of the relative abundance distribution [136] [141] [142]) and approaches to their estimation (e.g., Bayesian approaches that account for detectability based on repeat sampling; [143]) would facilitate meaningful comparisons among studies and regions where sampling equipment, depths, and timing are comparable. Community-level diversity metrics (i.e. beta diversity, in contrast to the within-community, alpha diversity presented in the present study) would elucidate species turnover and gradients in species turnover in space and time. Multivariate ordination methods such as principal components analysis (PCA) and multi-dimensional scaling (MDS) can be employed to visualize how the plankton, fish and environmental variables interact in multivariate phase space. In PCA, time series of major principal component scores can be used to examine the temporal dynamics of the multivariate trajectory. Canonical analysis methods such as redundancy analysis (RDA) and canonical correspondence analysis (CCA) can be used to evaluate the relationship between zooplankton or nekton communities and environmental factors indicative of ecosystem-level oceanographic processes (e.g., [144]).

### Modeling as information support tools for ecosystem-based management

Ultimately, to understand the drivers and role of biodiversity it will be necessary to integrate information about trophic linkages among taxa or groups, zooplankton and pelagic nekton life history characteristics, and physiological and population responses to environmental changes. The interpretation of observations and development of a mechanistic understanding of climate and anthropogenic forcing of zooplankton and fish biodiversity will likely require a synthesis among a wide range of modeling approaches, including population, integrative ecosystem, and food web modeling [145] [146]. Population dynamics modeling approaches, including coupled, 3-D physical-biological modeling, focus on the effect of climate forcing (e.g. variability in circulation, water temperature, and pH) on key species (e.g., [146] [147] [148] [149] [150]). Whole ecosystem models such as the Ecopath with Ecosim modelling tool (EwE), a mass-balance model from which temporal and spatial dynamic simulations can be developed, have been used in the northwest Atlantic and worldwide to quantitatively describe aquatic systems and to explore the ecosystem impacts of fishing, resiliency of ecosystems and component species, predator-prey dynamics, and food web complexity ([15] [16] [151] [152] [153] [154]). Similar mass-balance models for Georges Bank generally confirm EwE models [145]. A dynamic system model, ATLANTIS [155] [156], encompasses a virtual ocean with complex dynamics, a monitoring and assessment process, a set of ocean-uses (namely fishing), and a management process, and it will be used in the future to explore the likely effects of different management strategies on ecosystem processes. A suite of ‘minimum realistic’ models (MRMs) seek to explicitly add predation losses into single species assessment models of forage stocks, including Atlantic herring, Atlantic mackerel, longfin squid, and Northern shrimp [157] [158] [159] [160] [161]. Dynamics of multiple forage stocks that are both predators and prey of one another are also addressed by ‘extended’ multi-species virtual population analysis (MSVPA-X) [10] [162] [163]. Although zooplankton and pelagic nekton diversity is not explicit in any of these models, it is an emergent property under a wide range of environmental, climate, fishing, and predatory scenarios, and prediction and impacts of change in diversity can be assessed by integration of these modeling approaches.

### Conclusions

Substantial progress has been made in describing the diversity of plankton and pelagic nekton in the GoMA. The species list presented in Table S2, combined with the GoMRMS, represents the current state of knowledge of zooplankton and pelagic nekton species-level diversity in the region. Nevertheless, rare species from undersampled groups, for example deep-water gelatinous species, likely remain to be added. Spatial and temporal diversity patterns for mesozooplankton, particularly copepods, were identified where data from monitoring programs were available. Identification of diversity patterns is dependent on the availability of data with high taxonomic resolution, which currently are produced primarily through ship-board, net- or CPR-based sample collection and subsequent analysis by taxonomic specialists. The interdecadal changes in the copepod community observed in the CPR time series highlight the importance of sustained, consistent monitoring of the ecosystem.

The dynamic nature of the pelagic environment is reflected in spatial and temporal variability in the biomass and diversity of zooplankton species, which can serve as leading indicators of changing environmental or biological conditions. Knowledge of the relationship between diversity, environmental variability, and top-down control will contribute to prediction of how zooplankton and pelagic nekton diversity and ecological interactions will respond to environmental change and removals of zooplankton predators through fishing. While monitoring data were used here to describe spatial and temporal patterns of mesozooplankton diversity, they do not resolve the contributions of rare and undersampled taxa and groups to diversity. Alternative sampling methods will be required to identify the role of these species in the ecosystem, as well as to evaluate the influence of population- and functional-group level diversity on the ecosystem and to understand how the attributes of certain key species, such as *Calanus finmarchicus*, might influence trophic interactions.

Biodiversity is clearly important to ecosystem stability and resilience, both of which are essential to developing and maintaining sustainable human activities in marine ecosystems. Thus, it is important to convey information regarding the state of ecosystem diversity at the species and functional levels to managers. Both the Department of Fisheries and Oceans, Canada and the National Marine Fisheries Service, USA have released ecosystem status reports [32] [164] that include the Scotian Shelf and GoMA. At present, these reports emphasize changes in the abundance of dominant species or of taxonomic or functional groups. Reporting diversity metrics in addition to abundance metrics would provide a more rounded monitoring approach, given the apparent sensitivity of diversity to interannual environmental changes described above. While diversity metrics themselves do not provide information about the causes of the change, they would serve as an indicator of changes in ecosystem processes in future ecosystem status reports.

## Supporting Information

Table S1 **Total metazoan diversity from the Gulf of Maine Register of Marine Species (GoMRMS) and metazoan diversity from plankton samples.**
(DOC)

Table S2 **Phylogenetic list of all planktonic species listed in the plankton databases analyzed in this paper.**
(XLS)
