# Supplementary material for: Biodiversity and Ecosystem Function in the Gulf of Maine: Pattern and Role of Zooplankton and Pelagic Nekton
Source: PLoS One. 2011 Jan 31;6(1):e16491. doi: 10.1371/journal.pone.0016491 (PMC3031589; doi:10.1371/journal.pone.0016491)
Supplement: Table S1 — Total metazoan diversity from the Gulf of Maine Register of Marine Species (GoMRMS) and metazoan diversity from plankton samples. (DOC) [file pone.0016491.s001.doc]

**Table S1. Total metazoan diversity from the Gulf of Maine Register of Marine Species (GoMRMS) and metazoan diversity from plankton samples.**

|  |  | **GoMRMS** | | **Plankton Sample Data** | | **Provisional Additions** |
| --- | --- | --- | --- | --- | --- | --- |
| **Phylum** | **Class** | **# Families** | **# Species** | **# Families** | **# Species** | **# Species** |
| Porifera | Calcarea | 3 | 3 |  |  |  |
|  | Demospongiae | 15 | 25 |  |  |  |
| Cnidaria | Anthozoa | 24 | 46 |  |  |  |
|  | Hydrozoa | 36 | 109 | 7 | 10 | 3 |
|  | Scyphozoa | 5 | 7 | 3 | 4 |  |
|  | Staurozoa | 3 | 8 |  |  |  |
| Ctenophora | Nuda |  |  | 1 | 1 | 1 |
|  | Tentaculata | 3 | 4 | 1 | 1 |  |
| Acoelomorpha | Acoela | 7 | 12 |  |  |  |
| Platyhelminthes | Cestoda | 4 | 4 |  |  |  |
|  | Monogenea | 11 | 22 |  |  |  |
|  | Trematoda | 12 | 14 |  |  |  |
|  | Turbellaria | 13 | 20 |  |  |  |
| Nemertina | Anopla | 3 | 14 |  |  |  |
|  | Enopla | 7 | 21 |  |  |  |
| Rotifera | Eurotatoria | 2 | 2 |  |  |  |
|  | Pararotatoria | 1 | 2 |  |  |  |
| Nematoda | Adenophorea | 9 | 21 |  |  |  |
|  | Nematoda incertae sedis | 1 | 1 |  |  |  |
|  | Secernentea | 4 | 6 |  |  |  |
| Cephalorhyncha | Nematomorpha | 1 | 1 |  |  |  |
|  | Priapulida | 1 | 1 |  |  |  |
| Acanthocephala | Eoacanthocephala | 2 | 2 |  |  |  |
|  | Palaeacanthocephala | 5 | 25 |  |  |  |
| Entoprocta | unclassified | 2 | 2 |  |  |  |
| Echiura | Echiuroidea | 1 | 1 |  |  |  |
| Sipuncula | Sipunculidea | 3 | 6 |  |  |  |
| Annelida | Clitellata | 5 | 26 |  |  |  |
|  | Polychaeta | 55 | 341 | 1 | 2 |  |
| Arthropoda | Arachnida | 1 | 1 |  |  |  |
|  | Branchiopoda | 1 | 1 | 2 | 6 | 6 |
|  | Malacostraca | 92 | 285 | 25 | 63 | 30 |
|  | Maxillopoda | 50 | 113 | 41 | 159 | 118 |
|  | Merostomata | 1 | 1 |  |  |  |
|  | Ostracoda | 14 | 28 | 2 | 9 | 9 |
|  | Pycnogonida | 5 | 11 |  |  |  |
| Mollusca | Bivalvia | 39 | 114 |  |  |  |
|  | Caudofoveata | 1 | 2 |  |  |  |
|  | Cephalopoda | 4 | 7 |  |  |  |
|  | Gastropoda | 69 | 234 | 4 | 13 | 9 |
|  | Polyplacophora | 5 | 9 |  |  |  |
|  | Scaphopoda | 4 | 12 |  |  |  |
| Phoronida |  | 1 | 1 |  |  |  |
| Bryozoa | Gymnolaemata | 24 | 54 |  |  |  |
|  | Stenolaemata | 6 | 9 |  |  |  |
| Brachiopoda | Rhynchonellata | 1 | 1 |  |  |  |
| Echinodermata | Asteroidea | 8 | 20 |  |  |  |
|  | Echinoidea | 10 | 17 |  |  |  |
|  | Holothuroidea | 7 | 17 |  |  |  |
|  | Ophiuroidea | 6 | 8 |  |  |  |
| Chaetognatha | Sagittoidea | 2 | 5 | 2 | 12 | 7 |
| Hemichordata | Enteropneusta | 1 | 4 |  |  |  |
|  | Pterobranchia | 1 | 1 |  |  |  |
| Chordata | Ascidiacea | 9 | 36 |  |  |  |
|  | Larvacea | 2 | 2 | 2 | 3 | 1 |
|  | Thaliacea | 1 | 3 | 1 | 3 | 1 |
| Sub-Phylum Vertebrata: Pisces | Actinopterygii | 147 | 456 | 88 | 246 | 63 |
|  | Cephalaspidomorphi | 1 | 1 |  |  |  |
|  | Elasmobranchii | 16 | 45 | 1 | 1 |  |
|  | Holocephali | 1 | 1 |  |  |  |
|  | Myxini | 1 | 1 |  |  |  |
| Total |  | 769 | 2246 | 181 | 533 | 248 |

Provisional additions are species identified in plankton samples that were not in the GoMRMS.
